# Polyamine-mediated inhibition of ferroptosis contributes to geroprotection

**DOI:** 10.15698/cst2026.07.318

**Published:** 2026-07-03

**Authors:** Frank Madeo, Didac Carmona-Gutierrez, Guido Kroemer

**Affiliations:** 1 Institute of Molecular Biosciences, NAWI Graz, University of Graz, Graz 8010, Austria; 2 BioHealth Graz, Graz 8010, Austria; 3 BioTechMed Graz, Graz 8010, Austria; 4 Université Paris Cité, Sorbonne Université, Inserm U1138, Centre de Recherche des Cordeliers, Equipe labellisée par la Ligue contre le cancer, Paris, France; 5 Université Paris-Saclay, INSERM US23/CNRS UAR 3655, Metabolomics and Cell Biology Platforms, UMS AMMICa, Institut Gustave Roussy, Villejuif, France; 6 Institut du Cancer Paris CARPEM, Department of Biology, Hôpital Européen Georges Pompidou, AP-HP, Paris, France

**Keywords:** Aging, cell death, metabolism, spermidine, spermine, autophagy

## Abstract

Geroprotection aims at extending healthspan by delaying age-associated pathologies. Polyamines including spermine and spermidine are interconvertible metabolites whose longevity-promoting effects have traditionally been attributed to autophagy induction. In addition, recent evidence identifies spermine as an endogenous Fe^2+^ chelator that suppresses ferroptosis, thereby complementing the autophagy-inducing activity of spermidine. Indeed, spermidine inhibits EP300 acetyltransferase activity and supports hypusination-dependent activation of TFEB, both leading to autophagy. However, enhanced autophagic flux may increase susceptibility to ferroptosis through ferritinophagy and lipid remodeling. In parallel, polyamine catabolism generates H_2_O_2_ and acrolein, both of which facilitate lipid peroxidation and ferroptotic demise. The discovery that spermine directly chelates redox-active Fe^2+^ closes a conceptual gap by explaining how polyamine supplementation can promote longevity while avoiding excessive ferroptotic cell loss. Multiple lines of evidence including metabolomics, isotope tracing, cell-free lipid peroxidation systems, Fe^2+^-binding biophysics, mass spectrometry, Raman spectroscopy, nuclear magnetic resonance and disease models demonstrate that spermine limits labile iron and ferroptosis. Together, these findings support a unified model in which spermidine-driven autophagy and spermine-mediated ferroptosis inhibition cooperate to preserve tissue homeostasis and healthspan.

Geroprotection, the preservation of physiological fitness and prevention of age-related disease, has emerged as a central objective of modern biomedicine 1. This concept encompasses not only the extension of lifespan but also the prolongation of healthspan, namely the period of life spent free from chronic disease and functional decline.

**Figure 1:**
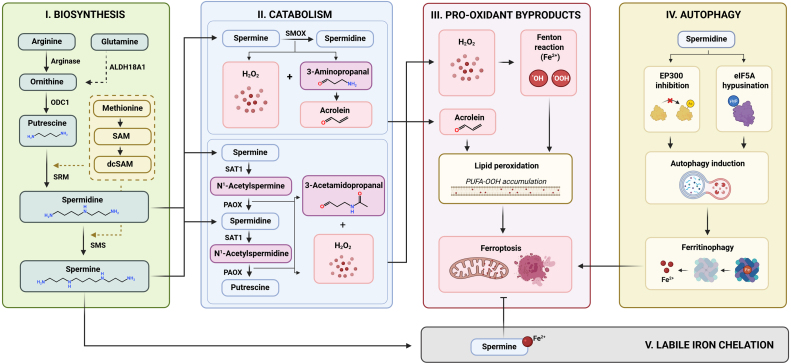
Polyamine biosynthesis and catabolism in mammals and their links to autophagy and ferroptosis. Polyamine biosynthesis begins with conversion of arginine to ornithine by arginase and subsequently to putrescine by ornithine decarboxylase (ODC1). Ornithine can also be generated from glutamine via an alternative pathway involving aldehyde dehydrogenase 18 family member A1 (ALDH18A1). Decarboxylated S-adenosylmethionine (dcSAM), generated from methionine via S-adenosylmethionine (SAM), provides aminopropyl groups for synthesis of spermidine and spermine by spermidine synthase (SRM) and spermine synthase (SMS), respectively. Polyamine catabolism occurs through two major pathways: (i) direct oxidation of spermine by spermine oxidase (SMOX) and (ii) acetylation by spermidine/spermine N^1^-acetyltransferase (SAT1) followed by oxidation by polyamine oxidase (PAOX). Both pathways generate hydrogen peroxide (H_2_O_2_) and 3-substituted propanals. 3-aminopropanal can be converted to the reactive aldehyde acrolein. H_2_O_2_ and acrolein promote oxidative stress and lipid peroxidation, leading to ferroptosis, an iron-dependent form of regulated cell death. Spermidine stimulates autophagy through inhibition of the acetyltransferase EP300 and by serving as precursor for eIF5A hypusination. Autophagy, particularly ferritinophagy, which *selectively* targets intracellular ferritin for degradation. increases the labile Fe^2+^ pool and thereby sensitizes cells to ferroptosis. Conversely, spermine may exert anti-ferroptotic effects by chelating or buffering redox-active Fe^2+^, reducing Fenton chemistry, lipid peroxidation, and ferroptotic cell death. The figure highlights the dual role of polyamine metabolism as both a promoter and inhibitor of ferroptosis through distinct biochemical mechanisms. Figure created in BioRender, https://BioRender.com/kxf9zze.

Among nutritional and pharmacological interventions, supplementation with the natural polyamines spermidine and spermine occupies a privileged position. Epidemiological, experimental and mechanistic studies have converged on the conclusion that both spermidine and spermine support healthy aging 2–5. Indeed, declining endogenous polyamine concentrations have been associated with aging in multiple tissues, suggesting that restoration of polyamine homeostasis may counteract fundamental aging mechanisms. The classical explanation for this phenomenon has been the capacity of spermidine to stimulate autophagy, a cytoprotective process that removes damaged proteins and organelles 6, 7. Yet a paradox has remained unresolved: autophagy can facilitate ferroptosis, an iron-dependent form of regulated necrosis driven by lipid peroxidation that is involved in several major diseases 8–12. This apparent contradiction has complicated efforts to integrate the pro-longevity effects of polyamines with the increasingly recognized role of ferroptotic cell death in age-associated pathology. The recent demonstration that spermine is a physiological Fe^2+^ chelator and ferroptosis inhibitor provides a compelling solution to this conundrum 13 (Figure 1).

Spermidine induces autophagy through at least two complementary mechanisms. First, it inhibits the acetyltransferase EP300, thereby favoring deacetylation of autophagy proteins and enhancing autophagic flux 14, 15. Second, spermidine serves as the precursor for hypusination of eukaryotic initiation factor 5 alpha (eIF5A)-related translational control pathways that ultimately stabilize transcription factor EB (TFEB), the master transcription factor coordinating lysosomal biogenesis and autophagy 16, 17. These pathways contribute to improved proteostasis, mitochondrial quality control and organismal longevity. In addition, spermidine-induced autophagy has been linked to enhanced stem-cell maintenance 18, improved immune function and memory during aging 19 as well as preservation of cardiovascular health in experimental models 2. However, autophagy is not unconditionally beneficial. Ferritinophagy, which is a special case of specific autophagy, liberates iron from ferritin, increasing the labile iron pool. Free Fe^2+^ then fuels Fenton reactions that facilitate irreversible oxidation and damage of cellular membranes, culminating in ferroptotic cell death 20. Moreover, ferroptosis is frequently accompanied by increased autophagic activity 21, and several forms of selective autophagy actively promote ferroptotic signaling 22, 23. Excessive ferroptosis may contribute to neurodegeneration, ischemia-reperfusion injury, inflammatory disorders and tissue aging 11, 24–26. Accordingly, the biological consequences of autophagy appear to depend on the cellular context and on the balance between adaptive recycling pathways and pro-death iron mobilization mechanisms.

Polyamine metabolism introduces an additional layer of complexity (Figure 1). Because spermidine and spermine undergo rapid metabolic interconversion, changes in one polyamine often affect the cellular levels of the other 27. Polyamine catabolism generates hydrogen peroxide and acrolein, potent pro-oxidant species that stimulate lipid peroxidation 28. These oxidative metabolites can damage membranes, proteins and nucleic acids, thereby amplifying cellular stress responses under conditions of excessive polyamine turnover 29. Thus, polyamine supplementation could theoretically increase ferroptotic risk by stimulating autophagy and by producing oxidative catabolites (Figure 1).

The recent study published in *Nature* resolves this apparent contradiction 13. Multiple convergent methods, including metabolomics, isotope tracing and orthogonal biophysical approaches, revealed that spermine directly binds Fe^2+^ with micromolar affinity. Spermine, but not spermidine or putrescine, reduced labile Fe^2+^, suppressed lipid peroxidation in cell-free systems, prevented ferroptotic death, and protected tissues from ischemia-reperfusion injury 13. Mass spectrometry identified spermine–Fe^2+^ complexes, while isothermal titration calorimetry, Raman spectroscopy and nuclear magnetic resonance confirmed direct coordination chemistry. Importantly, spermine lowered redox-active iron without significantly altering canonical ferroptosis regulators 13. These findings indicate that spermine acts upstream of many established ferroptotic pathways by directly restricting the availability of catalytically active iron required for lipid radical propagation (Figure 1).

Altogether, these observations suggest a division of labor within the polyamine network. Spermidine acts primarily as an autophagy inducer 2, 30, whereas spermine acts primarily as a ferroptosis suppressor 13. Because spermine and spermidine are metabolically interconvertible 27, administration of either compound may engage both protective modules. In this model, spermidine-driven autophagy rejuvenates the intracellular environment, while spermine-mediated Fe^2+^ sequestration prevents excessive ferroptotic cell loss. The result is a balanced enhancement of cellular housekeeping accompanied by preservation of cellular viability (Figure 1). Such a dual-action framework helps explain why polyamine supplementation consistently produces beneficial effects across diverse experimental systems despite the potentially hazardous consequences of excessive autophagic activation.

The conceptual implications extend beyond aging. Cancer, ischemia-reperfusion injury, neurodegeneration and chronic inflammatory diseases all involve complex interactions among autophagy, iron metabolism and oxidative stress 11, 24–26, 31. The identification of spermine as an endogenous iron chelator introduces a new metabolic checkpoint controlling ferroptosis. More broadly, these observations reinforce the notion that endogenous metabolites can function not merely as metabolic intermediates but also as direct regulators of cell fate decisions. From a geroscience perspective, the work suggests that successful longevity interventions may require the coordinated activation of adaptive stress responses together with protection against maladaptive cell death.

In conclusion, the emerging picture is remarkably coherent. Spermidine promotes longevity through autophagy induction, whereas spermine promotes longevity through ferroptosis inhibition. Their interconversion creates a dynamic polyamine network capable of simultaneously stimulating cellular renewal and restraining iron-dependent cell death. The discovery of spermine-mediated Fe^2+^ chelation therefore closes a major mechanistic gap in our understanding of polyamine-mediated geroprotection and strengthens the rationale for exploiting polyamines as interventions to extend human healthspan. Future studies must determine whether pharmacological modulation of the spermidine–spermine axis can be leveraged therapeutically to prevent age-related diseases and enhance resilience to cellular stress in humans.

## AUTHORS CONTRIBUTIONS

All authors participated to writing and editing of this paper.

## CONFLICT OF INTEREST

GK has been holding research contracts with Daiichi Sankyo, Eleor, Kaleido, Lytix Pharma, PharmaMar, Osasuna Therapeutics, Samsara Therapeutics, Sanofi, Sutro, Tollys, and Vascage. GK is on the Board of Directors of the Bristol Myers Squibb Foundation France. GK is a scientific co-founder of everImmune, Osasuna Therapeutics, Samsara Therapeutics, Sanvitalia and Therafast Bio. GK is in the scientific advisory boards of Hevolution, Institut Servier, and Rejuveron Life Sciences/Centenara Labs AG. GK is the inventor of patents covering therapeutic targeting of aging, cancer, cystic fibrosis and metabolic disorders. GK’s brother, Romano Kroemer, was an employee of Sanofi and now consults for Boehringer-Ingelheim. GK’s wife, Laurence Zitvogel, has held research contracts with Glaxo Smyth Kline, Incyte, Lytix, Kaleido, Innovate Pharma, Daiichi Sankyo, Pilege, Merus, Transgene, 9 m, Tusk and Roche, was on the on the Board of Directors of Transgene, is a cofounder of everImmune, and holds patents covering the treatment of cancer and the therapeutic manipulation of the microbiota. FM is an advisor for TLL The Longevity Labs GmbH..

## ABBREVIATIONS

ALDH18A1 – aldehyde dehydrogenase 18 family member A1

dcSAM – decarboxylated SAM

eiF5α – eukaryotic initiation factor 5 alpha

ODC1 – ornithine decarboxylase

PAOX – polyamine oxidase

SAM – S-adenosylmethionine

SAT1 – spermidine/spermine N-acetyltransferase

SMS – SMOXspermine synthase

SRM – spermidine synthase

TFEB – transcription factor EB
